# Cognitive and Reading Profiles of Gifted Students with Learning Disabilities: Implications for Assessment and Identification

**DOI:** 10.3390/bs16040599

**Published:** 2026-04-17

**Authors:** Susana Padeliadu, Athina Voulgari

**Affiliations:** School of Philosophy, Aristotle University of Thessaloniki, AUTH Campus, 54124 Thessaloniki, Greece; athinacv@edlit.auth.gr

**Keywords:** gifted with learning disabilities, dyslexia, cognitive profiles, reading profiles, twice-exceptionality

## Abstract

The identification of gifted students with learning disabilities (GLD) remains theoretically and methodologically contested. The present study examined cognitive and reading profiles of 150 Greek students in Grades 4–6, classified as gifted with learning disabilities (GLD) (*n* = 36), gifted (*n* = 31), or dyslexic of average intellectual ability (*n* = 83). Gifted classification was based on National Association for Gifted Children guidelines issued in 2018, using reasoning-based WISC-VIndices (FSIQ, GAI, EGAI, NVI, VECI ≥ 120), while learning disability was determined through formal multidisciplinary diagnosis. Cognitive performance was assessed with the WISC-V and reading with the standardized DADA battery (decoding, fluency, and comprehension). One-way ANOVAs and ROC analyses were conducted. GLD students demonstrated reasoning abilities and processing speed abilities comparable to gifted peers, but working memory deficits compared to gifted peers. In reading, GLD students showed decoding deficits like dyslexic peers and fluency impairments indistinguishable from them, yet significantly stronger comprehension. These findings reveal a differentiated literacy profile in which higher-order reasoning appears to support meaning construction despite persistent efficiency-based constraints in decoding and fluency. Overall, the results indicate that twice-exceptionality reflects a structurally uneven cognitive–academic configuration, underscoring the importance of multidimensional assessment approaches that simultaneously evaluate reasoning strengths and reading-specific vulnerabilities.

## 1. Introduction

Students who simultaneously exhibit exceptional cognitive potential and significant academic difficulties—commonly described as gifted students with learning disabilities (GLD)—present a distinctive challenge for researchers and practitioners. Although the existence of twice-exceptional learners is widely acknowledged (McCallum et al., 2013; Reis et al., 2014; Ronksley-Pavia, 2015), consensus remains elusive regarding their conceptual foundations and identification criteria. From a theoretical perspective, twice-exceptionality unsettles long-standing assumptions about the coherence and unity of intelligence. Traditional psychometric paradigms grounded in g-centric models assume that high general intelligence should be accompanied by consistently strong academic performance. GLD profiles, however, reveal that advanced reasoning abilities may coexist with pronounced weaknesses in specific cognitive processes, thereby challenging linear ability–achievement models and unitary interpretations of intellectual functioning.

The formal recognition of twice-exceptional learners at the 1981 Johns Hopkins University conference marked a pivotal moment in the development of this field (Brody & Mills, 1997). Early descriptive studies emphasized the paradoxical nature of GLD profiles, documenting how advanced verbal reasoning, abstract thinking, and metacognition coexist with deficits in working memory, and processing speed (Hannah & Shore, 1995, 2008; Reis et al., 2000; Waldron & Saphire, 1990). Although these studies were often limited in sample size and methodological rigor, they established a foundational insight: twice-exceptionality cannot be adequately understood within models that assume uniform alignment between cognitive strengths and academic performance.

Subsequent comparative research refined this picture by directly contrasting GLD students with gifted and dyslexic peers. GLD students typically resemble dyslexic peers on efficiency-based tasks involving decoding, rapid naming, and working memory (Berninger & Abbott, 2013; van Viersen et al., 2015, 2016), supporting a dual-component conceptualization in which high-capacity reasoning operates alongside constrained processing efficiency. van Viersen et al. (2016) found that GLD students outperformed dyslexic peers of average ability on several literacy measures yet performed below gifted and typically developing students, and that nonword reading fluency did not reliably distinguish dyslexia within gifted populations. Their earlier work (van Viersen et al., 2015) introduced the concept of “borderline dyslexia,” illustrating how elevated cognitive ability can raise absolute performance above diagnostic thresholds despite persistent underlying deficits.

Large-scale identification studies have added further nuance to this picture. McCallum et al. (2013), Bell et al. (2015), Ottone-Cross et al. (2017), and Maddocks (2018, 2020) demonstrated that GLD profiles are more consistently identified when intra-individual discrepancies and processing-deficit criteria are incorporated into assessment models. Using the KTEA-3, Ottone-Cross et al. (2017) emphasized the importance of profile analysis and error patterns rather than reliance on cumulative achievement scores. Maddocks (2018), analyzing co-normed Woodcock-Johnson III cognitive and achievement data, compared multiple identification frameworks and concluded that both discrepancy and processing-deficit criteria should inform GLD screening. In a subsequent study, Maddocks (2020) operationalized giftedness using reasoning-related indices ≥120 and defined GLD status based on the co-occurrence of high cognitive ability, significant ability–achievement discrepancy, and processing weaknesses. Collectively, these findings underscore both the heterogeneity of GLD profiles and the methodological variability that continues to characterize the field.

A recurring explanatory construct across this literature is the phenomenon of masking and compensation. High reasoning abilities may obscure underlying deficits, leading to under-identification when only absolute achievement criteria are applied (Assouline et al., 2010; Bell et al., 2015; Jackson Gilman et al., 2013; Maddocks, 2018, 2020; Silverman & Gilman, 2020). Twice-exceptional and GLD students may be identified only as gifted, GLD students may be identified only as learning disabled and GLD students may be considered as typically developing because giftedness and learning disability mask each other (Atmaca & Baloğlu, 2022; Brody & Mills, 1997). Masking is conceptualized as a process whereby cognitive strengths elevate observable performance sufficiently to attenuate the overt expression of foundational weaknesses, while compensation refers to the hypothesized strategic use of higher-order reasoning to support performance in areas of deficit. These constructs are theoretically useful but should be understood as interpretive frameworks consistent with observed cross-sectional profiles, rather than as directly demonstrated causal mechanisms. They help explain why GLD students may not meet traditional diagnostic thresholds despite experiencing significant academic strain.

Review contributions have traced this conceptual evolution. Vaughn (1989) initially noted the appeal but empirical scarcity of GLD research; Cohen and Vaughn (1994) observed that many GLD students are first identified through LD evaluations rather than gifted screening. Lovett and Sparks (2011) argued that, regardless of definitional thresholds, a subset of students will meet criteria for co-occurring giftedness and learning disability, although the population remains small. Atmaca and Baloğlu (2022) found that GLD students performed lower than gifted students regarding FSIQ, Working Memory and Processing Speed, suggesting that when giftedness identification relies solely on FSIQ, some GLD students may not be identified due to lower-level deficits. More recently, Kranz et al. (2024) synthesized research on gifted students with dyslexia and proposed identification criteria distinguishing empirically supported strengths and weaknesses from more tentative indicators, particularly within less transparent orthographies. Prevalence estimates reflect this complexity: the International Dyslexia Association (2020) places giftedness with dyslexia at approximately 2–5% of the general population, whereas Gilger and Hynd (2008) estimated 1–3% for gifted students with reading disabilities.

Identification practices further complicate the landscape. The use of rigid IQ thresholds (e.g., ≥130) for gifted identification has been criticized by advocates for twice-exceptional learners (Assouline et al., 2010; Jackson Gilman et al., 2013). Alternative approaches have proposed lower cutoffs (e.g., ≥120), reliance on the General Ability Index (GAI), or domain-specific identification based on elevated Verbal Comprehension or Perceptual Reasoning Indices of Wechsler scales. The National Association for Gifted Children (2018) supports the use of such alternative indices when working memory or processing speed weaknesses depress global IQ scores.

Taken together, these strands of research converge on a central conclusion: giftedness and learning disability intersect within complex, uneven cognitive configurations. Elevated reasoning ability may alter the manifestation of learning disability through compensatory mechanisms, while efficiency-based weaknesses may attenuate the observable expression of giftedness. This interpretation aligns with CHC theory, which differentiates broad reasoning abilities (Gf, Gc) from cognitive efficiency factors such as processing speed (Gs) and working memory (Gwm) (Schneider & McGrew, 2018). Within such a framework, uneven cognitive profiles are not anomalous but theoretically plausible outcomes of partially independent cognitive subsystems. The WISC-V, with its differentiated five-factor structure separating verbal comprehension, visual–spatial ability, fluid reasoning, working memory, and processing speed, provides a theoretically coherent instrument for examining such profiles. When interpreted in accordance with National Association for Gifted Children (2018) guidelines—emphasizing the GAI, EGAI, NVI, and VECI—it permits a more precise evaluation of high-level reasoning independent of efficiency-based weaknesses.

Empirical studies have inconsistently applied modern cognitive assessment tools, including the WISC-V, in alignment with contemporary identification guidelines such as those proposed by the National Association for Gifted Children (2018). Moreover, research on reading in twice-exceptional students has predominantly emphasized decoding accuracy and phonological deficits, with comparatively less attention to fluency and comprehension (Kranz et al., 2024). Because reasoning strengths may facilitate higher-order meaning construction despite foundational weaknesses, multidimensional literacy assessment is necessary to capture the full academic profile of GLD students. Finally, few studies have employed discriminative statistical approaches, such as Receiver Operating Characteristic (ROC) analyses, to evaluate the discriminative utility of cognitive and academic indices.

A further methodological consideration concerns the orthographic context of the present study. Greek is widely classified as one of the most transparent alphabetic orthographies for reading, with highly consistent grapheme–phoneme correspondences (Protopapas & Vlahou, 2009; Seymour et al., 2003). Therefore, implications may exist for how GLD students are identified and how their reading difficulties manifest. Because decoding accuracy is less diagnostically sensitive in Greek than in opaque orthographies such as English, fluency measures may carry particular weight in identifying reading disabilities, especially in gifted populations.

### The Present Study

The present study addresses these conceptual and methodological gaps by examining the cognitive and reading profiles of gifted students, students with dyslexia of average intellectual ability, and gifted students with learning disabilities (GLD) within the Greek educational context. Using WISC-V Indices aligned with National Association for Gifted Children (2018) recommendations and a comprehensive standardized reading battery (DADA), the study pursues three objectives:

1. To compare cognitive profiles across groups with particular attention to reasoning-based and efficiency-based indices.
2. To examine reading performance across decoding, fluency, and comprehension.
3. To evaluate the discriminative utility of cognitive and reading measures using Receiver Operating Characteristic (ROC) analyses.

The study employed a cross-sectional comparative design to examine cognitive and reading differences among three groups: gifted students with learning disabilities (GLD), gifted students, and students with dyslexia of average intellectual ability. The design integrated standardized cognitive and academic assessment data obtained through formal psychoeducational evaluation procedures within the Greek educational system.

By integrating contemporary identification guidelines with multidimensional assessment procedures, the study seeks to refine theoretical understanding of twice-exceptionality and contribute empirically grounded insights to psychoeducational evaluation.

## 2. Materials and Methods

### 2.1. Participants

Participants were 150 students enrolled in Grades 4 through 6 who had undergone psychoeducational evaluation using the WISC-V (Motibo, Athens, Greece) and the standardized Greek reading battery DADA (Rocket-Lexia, Thessaloniki, Greece). Participants were assigned to one of three groups based on predefined cognitive and diagnostic criteria, applied independently of the comparative analyses. The **gifted group** (*n* = 31) was recruited through collaboration with organizations supporting gifted education and through targeted public announcements. Classification as gifted was operationalized using criteria consistent with recommendations of the National Association for Gifted Children (2018). Specifically, students were required to obtain a score of ≥120 on the Full Scale IQ Score (FSIQ) and/or at least one reasoning-based WISC-V Index minimally influenced by working memory and processing speed, including the General Ability Index (GAI), the Expanded General Ability Index (EGAI), the Nonverbal Ability Index (NVI) or the Verbal Expanded Crystallized Index (VECI). This approach prioritizes higher-order reasoning abilities while reducing potential suppression effects from efficiency-related weaknesses. Participants in this group had no documented history or formal diagnosis of Specific Learning Disability (SLD) or other neurodevelopmental conditions affecting academic achievement.

The **dyslexic group** (*n* = 83) comprised students who had received a formal diagnosis of Specific Learning Disability in reading (dyslexia) through KE.D.A.S.Y., Greece’s public diagnostic centers. Diagnosis followed standardized psychoeducational evaluation procedures and required (a) documented normative underachievement in reading-related measures, (b) multidisciplinary clinical consensus, and (c) evidence of deficits in one or more theoretically established cognitive correlates of dyslexia, including phonological awareness, rapid automatized naming, working memory, and orthographic processing. To ensure conceptual separation from giftedness, inclusion additionally required overall cognitive performance within the average range on the WISC–V, thereby excluding students meeting gifted-level thresholds.

The **gifted with learning disabilities (GLD) group** (*n* = 36) was identified through retrospective review of KEDASY clinical records. Inclusion required the simultaneous presence of two independently established criteria: (a) a formal diagnosis of Specific Learning Disability in reading, determined prior to the present study through normative underachievement and multidisciplinary consensus; and (b) gifted-level cognitive performance, defined as a score of ≥120 (or ≥130 where applicable) on at least one NAGC-recommended reasoning-based WISC-V Index (FSIQ, GAI, EGAI, NVI, and VECI). Importantly, classification was based on documented diagnostic decisions and standardized test scores obtained during routine psychoeducational evaluations, rather than on post hoc statistical clustering within the current dataset. This dual-criterion approach ensured that GLD status reflected both formally verified learning disability and elevated reasoning ability, consistent with contemporary theoretical models of twice-exceptionality emphasizing the coexistence of high-level reasoning and efficiency-based weaknesses.

By operationalizing group membership through predefined and theoretically grounded criteria prior to comparative analyses, the study minimized classification circularity and strengthened construct validity. This procedure allowed subsequent analyses—including ROC evaluations—to examine discriminative patterns among structurally defined groups rather than statistically derived categories. It should nevertheless be acknowledged that several WISC-V Indices used for classification (GAI, EGAI, NVI, and VECI) were also included in the comparative analyses. This partial overlap introduces a degree of criterion-related circularity: group differences on these specific indices are partly expected by design and should not be interpreted as fully independent findings. Analyses of indices not used for classification (e.g., VCI, VSI, FRI, WMI, PSI, and all subtest-level measures) provide additional uncontaminated evidence of the profile differences observed.

Although some of the WISC–V Indices used for classification were also examined analytically, the study focused on broader profile patterns across multiple cognitive and reading measures rather than relying on a single defining score. This approach supports the interpretive validity of the findings by situating group differences within multidimensional performance profiles rather than within isolated indices. Additionally, while recruitment pathways differed across groups—reflecting the practical realities of identifying gifted and clinically referred students—standardized assessment procedures and consistent inclusion criteria were applied to all participants to promote comparability across groups.

#### Demographic Characteristics

Participants included students from Grades 4, 5, and 6, with representation across genders. Group distributions are presented in Table 1. Preliminary analyses confirmed no statistically significant demographic differences across groups that would confound cognitive or reading comparisons.

### 2.2. Instruments

#### 2.2.1. Wechsler Intelligence Scale for Children—Fifth Edition (WISC-V)

The WISC-V was standardized in Greece (Stogiannidou et al., 2017) and provides a multidimensional assessment of cognitive functioning across five Primary Indices: the Verbal Comprehension Index (VCI), the Visual Spatial Index (VSI), the Fluid Reasoning Index (FRI), the Working Memory Index (WMI) and the Processing Speed Index (PSI) as well as the FSIQ. Ancillary Indices, including the General Ability Index (GAI), the Expanded General Ability Index (EGAI), the Nonverbal Index (NVI), and the Verbal Expanded Crystallized Index (VECI), were examined due to their relevance for twice-exceptional assessment. These indices reduce the influence of working memory and processing speed on overall cognitive evaluation. It is important to mention that the Expanded General Ability Index (EGAI) and the Verbal Expanded Crystallized Index (VECI) are Ancillary Indices that were developed based on the American standardization sample to facilitate a broad assessment of intellectual abilities and giftedness identification (Raiford et al., 2015; Raiford et al., 2019). Finally, Subscale-level scores were also analyzed to examine intra-individual variability.

#### 2.2.2. Greek Standardized Reading Assessment (DADA)

To assess reading we used a standardized reading test in Greek student population between Grade 1 and Grade 9 DADA (Padeliadu et al., 2019). The scale assesses different dimensions of reading, decoding, fluency and comprehension. Decoding includes pseudoword decoding, word decoding, real word identification and word recognition. The sum of the scores on the four aspects of decoding create a total decoding score. The reliability of the total decoding scale for the primary school students’ population was ω = 0.895. Fluency is measured by correct words read orally by a text for one minute and comprehension involves answering multiple choice questions, based on texts read by the student. The reliability of the comprehension scale for the primary school students’ population was ω = 0.893. A total score is estimated by the student’s performance on decoding, fluency and comprehension. The performance on each dimension of reading is registered by raw scores, percentile scores and z scores. It should be noted that the DADA battery yields composite standardized scores for each reading dimension; its standardized scoring protocol does not enable systematic error-type or qualitative strategy analysis.

### 2.3. Data Analysis

Group differences in cognitive and reading performance were examined using one-way analyses of variance (ANOVAs). Where the homogeneity of variance assumptions was violated, Dunnett’s T3 post hoc comparisons were applied. Effect sizes were calculated using partial eta squared (η^2^) for omnibus tests and Cohen’s d for pairwise comparisons. Given the large number of pairwise comparisons reported across multiple indices (Table 2, Table 3 and Table 4), Bonferroni correction was applied to control for Type I error inflation. The corrected significance threshold was set at α = 0.05 divided by the number of comparisons per table. Comparisons that meet the corrected threshold are reported as statistically significant.

Receiver Operating Characteristic (ROC) analyses were conducted to evaluate the discriminative accuracy of cognitive and reading indices. Area Under the Curve (AUC) values were interpreted according to conventional standards (0.70 = acceptable, 0.80 = good, and 0.90 = excellent). Confidence intervals were calculated to assess statistical precision.

### 2.4. Ethical Considerations

All data were originally collected as part of standard psychoeducational evaluations conducted by public agencies. The study received approval from the Ethics Committee of the Department of Philosophy and Education of Aristotle University of Thessaloniki and from the Institute of Educational Policy of the Greek Ministry of Education. All identifying information was removed prior to analysis. Participation was voluntary, and parents were informed about the research purposes through KEDASY personnel. The study adhered to ethical standards concerning confidentiality, anonymity, and responsible data use.

## 3. Results

The results are organized according to the three study objectives: (a) cognitive differences across groups, (b) reading performance differences, and (c) discriminative accuracy of cognitive and reading indices. Preliminary screening confirmed that assumptions for parametric analyses were met; where homogeneity of variance was violated, Dunnett’s T3 corrections were applied. Effect sizes are reported using partial eta squared (η^2^) for omnibus tests and Cohen’s *d* for pairwise comparisons.

### 3.1. Cognitive Profiles Across Groups (WISC-V)

One-way ANOVAs revealed significant group differences across all five Primary WISC-V Indices and the FSIQ (all *p*s < 0.001), with effect sizes ranging from small-to-moderate (PSI: η^2^ = 0.146) to large (FSIQ: η^2^ = 0.779). Primary Index statistics were: VCI, *F*(2, 147) = 85.48, *p* < 0.001, η^2^ = 0.538; VSI, *F*(2, 147) = 80.20, *p* < 0.001, η^2^ = 0.522; FRI, *F*(2, 147) = 99.84, *p* < 0.001, η^2^ = 0.576; WMI, *F*(2, 147) = 61.97, *p* < 0.001, η^2^ = 0.457; PSI, *F*(2, 147) = 12.60, *p* < 0.001, η^2^ = 0.146; and FSIQ, *F*(2, 147) = 259.75, *p* < 0.001, η^2^ = 0.779. Significant differences were also observed across all four Ancillary Indices (all *ps* < 0.001): FSIQ, *F*(2, 147) = 259.75, η^2^ = 0.779; GAI, *F*(2, 147) = 200.89, η^2^ = 0.732; EGAI, *F*(2, 147) = 212.26, η^2^ = 0.743; NVI, *F*(2, 147) = 153.18, η^2^ = 0.676; and VECI, *F*(2, 147) = 99.69, η^2^ = 0.576.

Pairwise comparisons showed that GLD students performed at levels statistically indistinguishable from gifted peers on reasoning-related indices, including GAI, EGAI, NVI and VECI (all *p* > 0.05) but not FSIQ (*p* = 0.008). In contrast, GLD students scored significantly higher than dyslexic students on all reasoning indices (all *p* < 0.001), with large effect sizes (Cohen’s d range: 1.88–3.80; see Table 2 for full pairwise statistics).

A different pattern emerged for efficiency-based indices (Table 2). Working Memory Index (WMI) scores significantly differentiated all three groups. GLD students performed significantly below gifted peers (*p* < 0.001, Cohen’s d = −1.29), yet significantly above dyslexic peers (*p* < 0.001, Cohen’s d = 0.83), reflecting an intermediate profile. Processing Speed Index (PSI) showed a different configuration: GLD students outperformed the dyslexic group (*p* < 0.05, Cohen’s d = 0.49) but did not significantly differ from the gifted group.

Subscale analyses revealed pronounced intra-individual variability within the GLD group. GLD students performed at levels comparable to gifted peers on reasoning-intensive subtests such as Similarities, Vocabulary, Block Design, Visual Puzzles Matrix Reasoning and Figure Weights, (all *p* > 0.05), but scored significantly lower than gifted peers on subtests requiring manipulation of information under time or memory constraints, including Digit Span (*p* < 0.001, Cohen’s d = −1.37), Letter–Number Sequencing (*p* < 0.001, Cohen’s d = −1.82), Arithmetic (*p* = 0.002, Cohen’s d = −0.89), and Picture Span (*p* = 0.023, Cohen’s d = −0.68) (all *p*s < 0.05) (Table 3). Relative to dyslexic peers, GLD students scored significantly higher on all reasoning-intensive subtests (all *p*s < 0.001, Cohen’s d range: 1.50–1.99) and on several efficiency-dependent measures—Digit Span, Picture Span, Letter–Number Sequencing, and Coding (all *p*s < 0.05, Cohen’s d range: 0.47–0.78; see Table 3). However, GLD students’ performance on these same working memory subtests remained significantly below that of gifted peers (all *p*s < 0.05). Collectively, these results indicate a hybrid cognitive configuration characterized by elevated conceptual reasoning and intermediate cognitive efficiency.

### 3.2. Reading Profiles Across Groups (DADA)

Reading performance was examined using standardized z-scores across decoding, fluency, and comprehension, and results are shown in Table 4.

Significant group differences were observed across all four decoding measures (all *p*s < 0.001; full statistics in Table 4): pseudoword decoding, *F*(2, 147) = 34.06, η^2^ = 0.317; word decoding, *F*(2, 147) = 59.58, η^2^ = 0.448; real word identification, *F*(2, 147) = 15.28, η^2^ = 0.172; and total decoding, *F*(2, 147) = 55.03, η^2^ = 0.428. Gifted students demonstrated the highest decoding performance, while dyslexic students showed marked deficits across decoding measures (Table 4). GLD students scored significantly below gifted peers (pseudoword decoding: *p* < 0.001, Cohen’s d = −1.64; word decoding: *p* < 0.001, Cohen’s d = −2.00; real word identification: *p* = 0.007, Cohen’s d = −0.76; total decoding: *p* < 0.001, Cohen’s d = −1.77) but did not differ significantly from dyslexic peers on decoding indices.

Reading fluency showed the clearest convergence between GLD and dyslexic groups. A significant group effect was observed, *F*(2, 147) = 84.995, *p* < 0.001, η^2^ = 0.536. Gifted students significantly outperformed GLD students (*p* < 0.001, Cohen’s d = −2.36), whereas GLD and dyslexic students did not significantly differ from one another (Table 4). This finding reveals that reading fluency remains substantially constrained in GLD students, paralleling the dyslexic profile, despite their good levels of processing speed. This finding is orthographically significant; fluency is the primary locus of reading difficulty in transparent orthographies such as Greek, where slow decoding is the hallmark of dyslexia (Diamanti et al., 2018; Douklias et al., 2009). Given that GLD students in the present study possessed strong verbal reasoning and vocabulary, reflected in elevated VCI and VECI scores, one might expect a fluency advantage. The absence of such an advantage reinforces the view that fluency constraints in GLD students are rooted in phonological automaticity, even within a highly transparent orthography.

Reading comprehension revealed a distinct pattern *F*(2, 147) = 26.619, *p* < 0.001, η^2^ = 0.266). GLD students performed significantly better than dyslexic students (*p* = 0.001, Cohen’s d = 0.67) but significantly lower than gifted students (*p* = 0.010, Cohen’s d = −0.77) (Table 4). Importantly, their comprehension performance exceeded what might be predicted based solely on their decoding and fluency weaknesses. This intermediate yet elevated comprehension profile is consistent with partial compensation through higher-order reasoning abilities.

### 3.3. Discriminative Accuracy (ROC Analyses)

Receiver Operating Characteristic (ROC) analyses were conducted to evaluate the discriminative utility of working memory and processing speed, to discriminate the three groups (gifted students, students with dyslexia and average intellectual ability, and GLD students). These analyses are reported as exploratory indicators of relative discriminative utility and are not intended to constitute or validate a clinical identification algorithm. Area Under the Curve (AUC) values were interpreted using conventional criteria (0.70 = acceptable, 0.80 = good, 0.90 = excellent).

**Figure 1:**
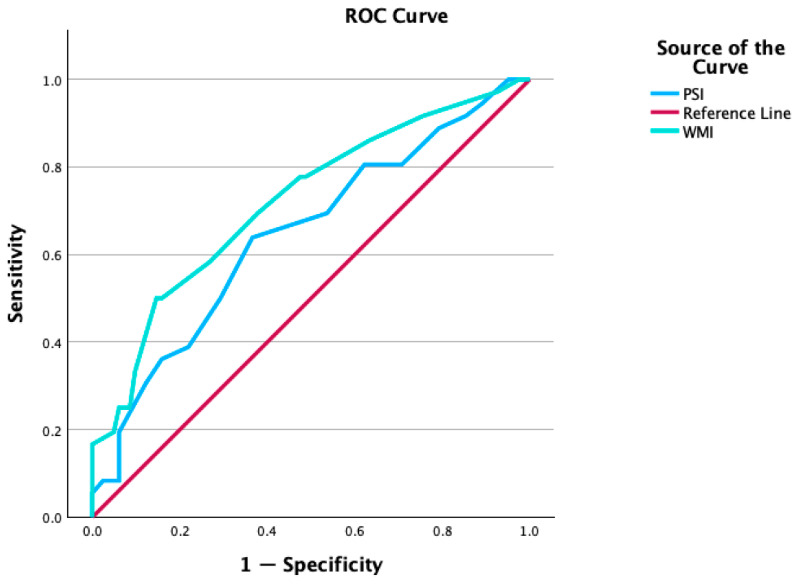
ROC curves for Working Memory (green line) and Processing Speed (blue line) for discriminating GLD students from students with dyslexia.

For distinguishing GLD students from students with dyslexia, working memory yielded an AUC of 0.72, 95% CI [0.62, 0.82], *p* < 0.001, indicating acceptable diagnostic accuracy (Hosmer et al., 2013). Processing speed yielded an AUC of 0.65, 95% CI [0.54, 0.76], *p* < 0.001, showing poor discrimination (Hosmer et al., 2013). The ROC curves for the two indicators are presented in Figure 1. Visual inspection of the curves confirms that working memory offers the best trade-off between sensitivity and specificity to discriminate gifted students with dyslexia from students with dyslexia.

**Figure 2:**
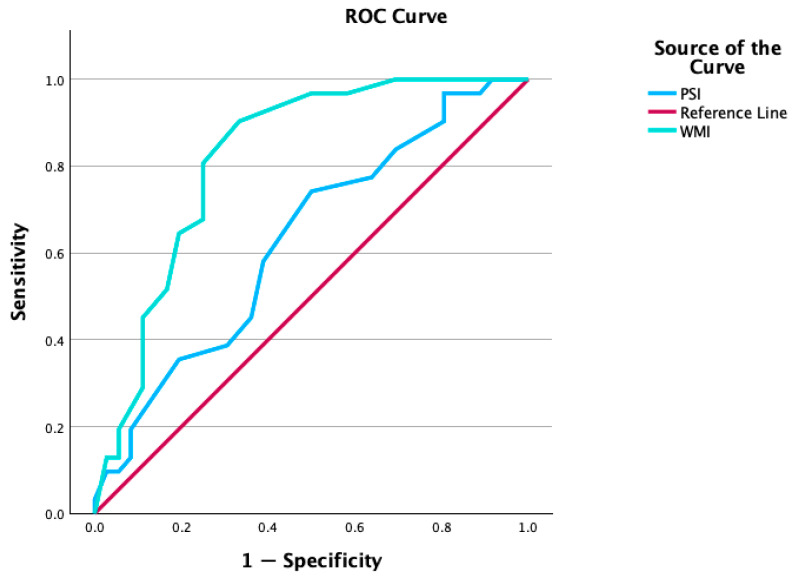
ROC curves for Working Memory (green line) and Processing Speed (blue line) for discriminating GLD students from gifted students.

For distinguishing GLD students from gifted students, working memory had an AUC of 0.82, 95% CI [0.72, 0.92], *p* < 0.001, indicating strong discrimination (Hosmer et al., 2013). Processing speed had an AUC of 0.63, 95% CI [0.50, 0.76], *p* < 0.001, indicating poor accuracy (Hosmer et al., 2013). Figure 2 visualizes the ROC curves for both indicators. Visual inspection of the ROC curves indicates that working memory provides the most favorable balance between sensitivity and specificity in distinguishing GLD students from gifted students.

To sum up, working memory consistently emerged as the relatively strongest cognitive indicator for distinguishing across gifted students, students with dyslexia, and GLD students. In contrast, processing speed showed limited discriminative utility across both comparisons.

## 4. Discussion

The present study examined the cognitive and reading profiles of gifted students with learning disabilities (GLD) in comparison with gifted peers without disabilities and dyslexic peers of average intellectual ability. By applying WISC-V Indices aligned with National Association for Gifted Children (2018) guidelines and incorporating a multidimensional reading assessment, the study offers novel empirical evidence concerning the nature of twice-exceptionality and the implications for psychoeducational assessment. The findings contribute to an expanding body of research (e.g., Hannah & Shore, 1995, 2008; Maddocks, 2018, 2020; Reis et al., 2000; van Viersen et al., 2015, 2016) by empirically illustrating how elevated reasoning abilities and efficiency-based weaknesses coexist within the same individuals.

### 4.1. Hybrid Cognitive Architecture

Consistent with prior comparative studies (Berninger & Abbott, 2013; Ferri et al., 1997; van Viersen et al., 2016; Waldron & Saphire, 1990), GLD students in the present study resembled gifted peers on reasoning-based indices (GAI, VECI, and NVI), while demonstrating working memory weaknesses more closely aligned with dyslexic peers. This pattern supports earlier observations that GLD profiles reflect dissociations between higher-order reasoning and foundational processing efficiency (Hannah & Shore, 1995; Maddocks, 2018; Reis et al., 2000).

These findings are theoretically coherent within multidimensional models of intelligence such as CHC theory (Schneider & McGrew, 2018), which distinguish broad reasoning abilities (e.g., Gf and Gc) from cognitive efficiency factors such as working memory (Gwm) and processing speed (Gs). Τhe key findings (working memory differentiation, fluency convergence, and comprehension advantage) remain significant after correction and are interpreted with appropriate caution. The pronounced intra-individual variability observed in GLD profiles further supports critiques of global IQ interpretation in twice-exceptional populations. As suggested by prior scholarship (Assouline et al., 2010; Atmaca & Baloğlu, 2022; Maddocks, 2018), aggregation of reasoning strengths and processing weaknesses into a single Full Scale IQ Score may obscure meaningful structural variability.

The ROC findings indicate that working memory is the relatively strongest discriminating cognitive variable for distinguishing twice-exceptional students from both gifted and dyslexic peers. When differentiating GLD students from those with dyslexia, working memory demonstrated acceptable accuracy (AUC = 0.72), suggesting that although both groups share decoding-related weaknesses, GLD students retain relatively stronger executive resources. More strikingly, working memory showed good discrimination between GLD and gifted students (AUC = 0.82), highlighting that the defining vulnerability in twice-exceptionality is not diminished reasoning but constrained executive efficiency. This pattern aligns with prior research demonstrating dissociations between higher-order reasoning and foundational processing capacities in GLD populations (Hannah & Shore, 1995; Waldron & Saphire, 1990; van Viersen et al., 2016; Maddocks, 2018). The notable overlap implied by an AUC of 0.72, however, confirms that working memory alone cannot serve as a definitive classifier. In contrast, processing speed demonstrated limited discriminative utility across both comparisons. Because processing speed weaknesses are frequently observed in learning disabilities (Atmaca & Baloğlu, 2022; Berninger & Abbott, 2013; Maddocks, 2020), they appear to lack the specificity needed to identify a twice-exceptional profile. Importantly, even the strongest discriminator did not reach good-to-excellent accuracy, confirming that no single index is sufficient for classification. These results reinforce calls for profile-level interpretation and multidimensional assessment when evaluating twice-exceptional learners, particularly where compensatory mechanisms may mask underlying vulnerabilities (Rizzo et al., 2025; Townend et al., 2024).

### 4.2. Reading Profiles: Differential Compensation Across Components

GLD students demonstrated decoding and fluency performance comparable to dyslexic peers yet inferior to gifted peers. Reading comprehension, in contrast, was stronger than that of dyslexic peers and approached—but did not match—that of gifted students. Importantly, the comprehension advantage observed in GLD students may be particularly pronounced—or more readily detected—in transparent orthographies such as Greek compared to opaque orthographies where decoding difficulties impose a heavier downstream drag on comprehension. Moreover, such findings are theoretically compatible with dual-route and capacity-limited models of reading, which distinguish between proceduralized decoding and higher-order semantic integration. These findings may also be understood in relation to the orthographic characteristics of Greek. The van Viersen et al. (2016) study was conducted in Dutch, a moderately transparent orthography, and found that GLD students outperformed dyslexic peers on reading fluency, suggesting that reading fluency should not be used as a valid criterion for detecting dyslexia especially in gifted students. The convergence of GLD and dyslexic students on fluency in the present study is precisely what orthographic transparency theory would predict: in a highly consistent orthography, reading speed and not only reading accuracy becomes a significant site of impairment (Diamanti et al., 2018). Accordingly, the present findings represent an orthography-specific replication and extension of van Viersen’s results, situating GLD reading profiles within the broader cross-linguistic literature and suggesting that the diagnostic weight of fluency measures may be elevated in transparent orthographic contexts.

Although our selection criteria guaranteed high reasoning scores, they did not mandate high reading comprehension. The finding that GLD students significantly outperformed dyslexic peers in comprehension—but not in fluency—is consistent with a partial compensation hypothesis—the possibility that intact Gc and Gf abilities support meaning construction despite persistent constraints in decoding and fluency—but should not be interpreted as direct evidence of compensatory processing, as the present cross-sectional design does not permit causal or process-level inference (Maddocks, 2020). Further, the cognitive profile observed in this study aligns closely with theoretical accounts of masking and compensation described in the literature (Assouline et al., 2010; Bell et al., 2015; Jackson Gilman et al., 2013; Maddocks, 2018, 2020; Silverman & Gilman, 2020). Elevated reasoning abilities may elevate observable academic performance, thereby reducing the likelihood that foundational weaknesses are detected through absolute achievement criteria. In this sense, the pattern is compatible with the view that masking reflects the attenuation of overt disability manifestation rather than its absence, a developmental hypothesis that longitudinal research would need to evaluate more directly.

## 5. Implications for Assessment

The findings have several implications for psychoeducational assessment. First, they highlight the limitations of relying solely on global IQ scores or isolated cognitive indicators when evaluating twice-exceptional learners (Atmaca & Baloğlu, 2022). Consequently, assessment practices that rely rigidly on standard cutoffs may fail to identify twice-exceptional learners. This supports the argument advanced by Lovett and Sparks (2011) that definitional thresholds alone are insufficient to resolve identification challenges; instead, profile-level interpretation is necessary. Second, these findings support the use of reasoning-based indices in twice-exceptional assessment. Reasoning indices can reveal high ability even when processing speed or working memory weaknesses depress composite scores, while efficiency indices can highlight areas of disability that may otherwise be overlooked. Third, reading assessment should extend beyond decoding accuracy to include fluency and comprehension, especially for older students (Kranz et al., 2024). GLD learners appear to struggle with decoding and fluency due to persistent cognitive constraints. Assessment batteries which include fluency and comprehension measures are better suited to capturing these complex profiles.

Beyond assessment, the findings highlight several considerations for classroom instruction and educational planning. GLD students require support that acknowledges both their strengths and weaknesses. Instructional approaches should challenge their reasoning abilities while simultaneously addressing their working memory limitations. For example, enrichment opportunities may be combined with targeted interventions in literacy fluency or executive functioning (Townend et al., 2024). Additionally, teachers should be aware that GLD learners may display uneven academic performance, fluctuating engagement, or signs of frustration that stem not from lack of motivation but from the cognitive tension between their strengths and weaknesses (Maddocks, 2020; Rizzo et al., 2025). The persistence of fluency difficulties, even among students with gifted-level reasoning, suggests that accommodations such as extended time, reduced reading load, or access to audiobooks may be beneficial. At the same time, educators should encourage the development of higher-order analytic and creative skills that leverage students’ exceptional abilities.

## 6. Limitations

Several limitations warrant consideration. First, the cross-sectional nature of the design precludes conclusions regarding longitudinal developmental trajectories or the stability of compensatory mechanisms over time. Second, although group classification followed formal diagnostic procedures and National Association for Gifted Children (2018) guidelines, partial overlap between identification criteria and cognitive indices examined may introduce some degree of criterion-related circularity. Analyses involving the primary classification indices (GAI, EGAI, NVI, and VECI) should be interpreted with this in mind, as group differences on these measures are partially expected by design. Future studies should separate classification and analysis indices more cleanly, either by using held-out validation samples or by examining indices not used for group formation. Moreover, the magnitude of the observed effect sizes between the groups could be influenced by the unequal group sizes. Bootstrapped confidence intervals for mean differences could be implemented in future replication studies. Third, recruitment procedures differed across groups—the gifted group was recruited through gifted education organizations and public announcements, while the dyslexic and the GLD group were recruited through retrospective KEDASY records. These differential pathways may introduce systematic referral biases: the GLD group may over-represent students with more clinically visible profiles, and the gifted group may reflect families with higher engagement with gifted education. This limits the generalizability of findings to population-representative samples. Fourth, the study did not collect data on socioeconomic status, parental education level, comorbid conditions, school type, or urban/rural location. These variables are well-established predictors of reading comprehension and cognitive development. Their omission means that observed group differences—particularly the comprehension advantage attributed to compensation in GLD students—cannot be fully disentangled from potential background effects. Future studies should incorporate SES and educational context as covariates. Fifth, the data reported from the DADA battery does not yield error-type or qualitative strategy data, precluding the kind of error-pattern analysis highlighted in prior GLD research (Ottone-Cross et al., 2017). This limits the depth of reading profile characterization possible within the current dataset. Sixth, the compensation interpretation is inferential rather than directly tested; the cross-sectional design cannot establish whether higher-order reasoning causally supports comprehension. Future studies using experimental or longitudinal designs with cognitive–linguistic mediation models would be needed to test this hypothesis directly. Further, the present study employed a theory-driven, a priori group comparison framework consistent with prior comparative research in this literature (e.g., Maddocks, 2018, 2020; van Viersen et al., 2015, 2016). Data-driven approaches such as latent profile analysis or discriminant function analysis represent complementary techniques that could be applied in future studies. Finally, the findings reflect assessment within a Greek orthographic context and may not generalize to languages with differing degrees of transparency. In more opaque orthographies such as English or French, GLD students may demonstrate more pronounced decoding accuracy advantages over dyslexic peers, while the relationship between reasoning-driven compensation and comprehension may interact differently with orthographic demands. Future research should examine whether the compensation pattern observed here—intact reasoning supporting comprehension despite persistent fluency constraints—replicates across orthographic systems of varying transparency.

## 7. Conclusions

Despite these limitations, the present study advances empirical and theoretical understanding of twice-exceptionality by integrating contemporary identification guidelines, multidimensional cognitive assessment, and comprehensive reading evaluation within a comparative framework. Consistent with prior research, GLD students demonstrated elevated reasoning abilities alongside efficiency-based weaknesses. The findings reinforce the need for profile-level interpretation in psychoeducational assessment and support the view that twice-exceptionality reflects an interactional cognitive configuration rather than the simple co-occurrence of two independent categories. 

## Figures and Tables

**Table 1:** Participant numbers, gender and grade per group.

	Gender		Class			Total
	Boys	Girls	4th Grade	5th Grade	6th Grade	
GLD	24	12	12	11	13	36
Gifted	15	16	11	9	11	31
Dyslexic of average intellectual ability	42	41	21	13	49	83

**Table 2:** Comparison of cognitive abilities across WISC-V Primary Indices, FSIQ, and Ancillary Indices.

	GLD		GF		DL		Group Comparisons			
Indices Not Used for Classification	Mean	SD	Mean	SD	Mean	SD	Difference 1 (GLD-GF)	Cohen’s d	Difference 2 (GLD-DL)	Cohen’s d
VCI	115.4	8.2	115.2	6.1	97.0	9.3	0.14		18.34 ***	1.88
VSI	122.3	9.5	120.1	8.8	101.7	9.6	2.24		−20.62 ***	1.99
FRI	119.6	6.7	119.8	6.8	101.6	8.5	−0.20		18.02 ***	2.08
WMI	93.5	12.8	107.9	9.3	84.2	9.3	−14.40 ***	−1.29	9.34 ***	0.83
PSI	105.1	11.9	111.0	12.5	99.2	10.8	−5.83		5.91 *	0.49
Indices used for classification										
FSIQ	116.8	5.8	120.8	4.7	96.2	6.6	−4.01 **	0.77	20.66 ***	3.01
GAI	121.3	5.1	121.5	6.0	99.3	7.5	−0.22		22.07 ***	3.80
EGAI	121.0	5.3	123.3	4.5	98.2	8.4	−2.29		22.76 ***	3.56
ΝVI	116.6	7.6	119.2	6.9	96.3	7.7	−2.52		20.33 ***	2.47
VECI	118.1	7.7	119.6	7.0	99.2	9.2	−1.51		18.95 ***	1.99

GLD: gifted with learning disability; GF: gifted; DL: dyslexic with average intellectual ability. * *p* < 0.05, ** *p* < 0.01, *** *p* < 0.001. WISC-Vabbreviations are: VCI: Verbal Comprehension Index; VSI: Visual Spatial Index; FRI: Fluid Reasoning Index; WMI: Working Memory Index; PSI: Processing Speed Index; FSIQ: Full Scale IQ Score; GAI: General Ability Index; EGAI: Expanded General Ability Index; NVI: Non Verbal Ability Index; VECI: Verbal Expanded Crystallized Index.

**Table 3:** Comparison of cognitive abilities across WISC-V scales.

	GLD		GF		DL		Group Comparisons			
Scale	Mean	SD	Mean	SD	Mean	SD	Difference 1 (GLD-GF)	Cohen’s d	Difference 2 (GLD-DL)	Cohen’s d
SI	13.5	1.8	13.4	1.3	9.8	2.1	0.08		3.66 ***	1.66
VC	12.2	1.8	12.1	1.7	9.0	1.8	0.09		3.22 ***	1.65
IN	13.4	1.9	13.6	2.2	9.6	2.5	−0.20		3.85 ***	1.94
CO	13.2	1.5	14.4	2.0	10.9	1.8	−1.19 *	.068	2.25 ***	1.20
BD	13.9	1.7	13.6	1.8	9.9	1.9	0.31		3.99 ***	1.99
VP	14.0	1.9	13.5	1.5	10.7	2.1	0.51		3.33 ***	1.53
MR	13.3	1.9	13.5	1.9	10.1	1.8	−0.17		3.18 ***	1.58
FW	13.4	1.6	13.4	1.3	10.5	1.9	0.09		2.96 ***	1.50
AR	11.7	1.9	13.5	2.2	8.2	2.2	−1.82 **	.089	3.51 ***	1.53
DS	9.4	2.3	12.5	2.2	7.7	2.1	−3.06 ***	1.37	1.70 ***	0.73
PS	8.4	2.8	10.2	2.6	6.9	2.0	−1.83 *	0.68	1.48 *	0.60
LNS	8.7	1.7	11.8	1.9	7.2	1.8	−3.17 ***	1.82	1.51 ***	0.78
CD	10.4	2.5	11.6	2.6	9.3	2.1	−1.20		1.12 *	0.47
SS	11.5	2.6	12.1	2.2	10.4	2.5	−0.63		1.11	
CA	11.8	2.0	12.8	2.4	11.1	2.1	−0.97		0.73	

GLD: gifted with learning disability; GF: gifted; DL: dyslexic with average intellectual ability. * *p* < 0.05, ** *p* < 0.01, *** *p* < 0.001. WISC-V abbreviations are: SI: Similarities; VC: Vocabulary; IN: Information; CO: Comprehension; BD: Block Design; VP: Visual Puzzles; MR: Matrix Reasoning; FW: Figure Weights; AR: Arithmetic; DS: Digit Span; PS: Picture Span; LNS: Letter–Number Sequencing; CD: Coding; SS: Symbol Search; CA: Cancelation.

**Table 4:** Comparison of reading performance (GLD, gifted and dyslexic of average intellectual ability).

Reading Dimension	GLD		GF		DL		Group Comparisons			
	Mean	SD	Mean	SD	Mean	SD	Difference 1 (GLD-GF)	Cohen’s d	Difference 2 (GLD-DL)	Cohen’s d
Pseudoword Decoding	−0.90	1.2	0.71	0.7	−1.3	1.3	−1.61 ***	−1.64	0.40	
Word Decoding	−0.67	0.76	0.89	0.82	−0.85	0.75	−1.56 ***	−2.00	0.18	
Real Word Identification	−0.01	0.92	0.61	0.69	−0.34	0.82	−0.62 **	−0.76	0.33	
Decoding (Total)	−0.76	0.95	0.90	0.96	−0.98	0.79	−1.66 ***	−1.77	0.22	
Fluency	−1.62	0.85	0.47	0.94	−1.94	0.88	−2.09 ***	−2.36	0.32	
Comprehension	0.56	0.89	1.18	0.70	−0.06	0.85	−0.61 *	−0.77	0.63 **	0.67

GLD: gifted with learning disability; GF: gifted; DL: dyslexic with average intellectual ability. * *p* < 0.05, ** *p* < 0.01, *** *p* < 0.001.

## Data Availability

Data is unavailable due to privacy and ethical restrictions. Further inquiries can be directed to the corresponding author.

## Abbreviations

The following abbreviations are used in this manuscript:

GLD	Gifted with learning disability
DL	Dyslexic with average intellectual ability
GF	Gifted
NAGC	National Association for Gifted Children
CHC theory	Cattell–Horn–Carroll theory
Gf	Fluid Reasoning
Gc	Verbal Comprehension
Gs	Processing Speed
Gwm	Working Memory
DADA	Standardized reading test in Greek population
KTEA-3	Kaufman Test of Educational Achievement--Third Edition (KTEA-3)
KEDASY	Greece’s public diagnostic centers
ROC	Receiver Operating Characteristic
VCI	Verbal Comprehension Index
VSI	Visual Spatial Index
FRI	Fluid Reasoning Index
WMI	Working Memory Index
PSI	Processing Speed Index
FSIQ	Full Scale IQ Score
GAI	General Ability Index
EGAI	Expanded General Ability Index
NVI	Non Verbal Ability Index
VECI	Verbal Expanded Crystallized Index
SI	Similarities
VC	Vocabulary
IN	Information
CO	Comprehension
BD	Block Design
VP	Visual Puzzles
MR	Matrix Reasoning
FW	Figure Weights
AR	Arithmetic
DS	Digit Span
PS	Picture Span
LNS	Letter–Number Sequencing
CD	Coding
SS	Symbol Search
CA	Cancelation
